# Systemic Treatment with Telmisartan Improves Femur Fracture Healing in Mice

**DOI:** 10.1371/journal.pone.0092085

**Published:** 2014-03-18

**Authors:** Xiong Zhao, Jia-xing Wang, Ya-fei Feng, Zi-xiang Wu, Yang Zhang, Lei Shi, Quan-chang Tan, Ya-bo Yan, Wei Lei

**Affiliations:** 1 Department of Orthopeadics, Xijing Hospital, The Fourth Military Medical University, Xi'an, PR China; 2 Department of Cardiology, Xijing Hospital, The Fourth Military Medical University, Xi'an, PR China; 3 ICU, 309th Hospital of PLA, Beijing, PR China; University of Toronto, Canada

## Abstract

Recent clinical studies indicated that angiotensin receptor blockers (ARBs) would decrease the risk of bone fractures in the elderly populations. There is little known about the role of the ARBs in the process of fracture healing. The purpose of the present study was to verify the hypothesis that systemic treatment with telmisartan has the ability to promote fracture healing. In this study, femur fractures were produced in 96 mature male BALB/c mice. Animals were treated with the ARBs telmisartan or vehicle. Fracture healing was analysed after 2, 5 and 10 weeks postoperatively using X-ray, biomechanical testing, histomorphometry, immunohistochemistry and micro-computed tomography (micro-CT). Radiological analysis showed the diameter of the callus in the telmisartan treated animals was significantly increased when compared with that of vehicle treated controls after two weeks of fracture healing. The radiologically observed promotion of callus formation was confirmed by histomorphometric analyses, which revealed a significantly increased amount of bone formation when compared with vehicle-treated controls. Biomechanical testing further showed a significantly greater peak torque at failure, and a higher torsional stiffness in telmisartan-treated animals compared with controls. There was an increased fraction of PCNA-positive cells and VEGF-positive cells in telmisartan-treated group compared with vehicle-treated controls. From the three-dimensional reconstruction of the bony callus, telmisartan-treated group significantly increased the values of BV/TV by 21.7% and CsAr by 26.0% compared to the vehicle-treated controls at 5 weeks post-fracture. In summary, we demonstrate in the current study that telmisartan could promote fracture healing in a mice model via increasing mechanical strength and improving microstructure. The most mechanism is probably by an increase of cell proliferation and neovascularization associated with a decreased VEGF expression in hypertrophic chondrocytes.

## Introduction

The renin–angiotensin system (RAS) is an endocrine system that controls body fluid and electrolyte balance and blood pressure [1]. The main effector peptide in this system is angiotensin II (Ang II), which is formed from angiotensin I (Ang I) by angiotensin-converting enzyme (ACE), a key molecule in this system. Angiotensin II (Ang II) acts via angiotensin II type 1 (AT1) and type 2 (AT2) receptors, which are members of the 7-transmembrane- spanning G-protein-coupled receptors. AT1 and AT2 receptors exhibit limited sequence homology (34% amino acid sequence identity) [2]. Many actions of Ang II appear to be through AT1 receptors. The RAS has been an important target of antihypertensive drugs, particularlly angiotensin-converting enzyme (ACE), inhibitors and angiotensin receptor blockers (ARBs) [3], [4].

Recent clinical studies indicated that beta blockers and anti-hypertension drugs would reduce the risk of bone fractures in the elderly populations [5]–[6]. This suggests a possible link between vascular and skeletal systems. Renin angiotensin system (RAS) is operating not only systemically but also locally in several tissues, and bone microenvironments have been studied in this regard [7]–[8]. In bone tissue, Ang II was reported to promote bone resorption via the AT1 receptor in cell culture system and in ovariectomized mice and rats [9]. Expression of AT1 receptor was observed in cultured osteoblasts, and Ang II inhibit differentiation and bone formation via the AT1 receptor in rat calvarial osteoblastic cells [10], [11].

A recent study showed that inhibition of angiotensin-converting enzyme has improved fracture healing and periosteal callus formation in a murine femur fracture model [12]. However, there is still little known about the role of the ARBs in the process of fracture healing. Therefore, the purpose of the present study was to verify the hypothesis that systemic treatment with telmisartan has the ability to promote fracture healing.

## Materials and Methods

### Animals

Ninety-six mature male BALB/c mice (purchased from the Experimental Animal Center of The Fourth Military Medical University, Xi'An, China) with an average weight of 24 g at the beginning of the study were used. The mice were kept five per cage with free access to mouse chow and water ad libitum. All experimental procedures of animal were approved by the Ethics in Animal Research Committee of The Fourth Military Medical University.

### Fracture surgery

The mice were anesthetized by i.p. injection of xylazine (25 mg/kg BW) and ketamine (75 mg/kg BW). Under sterile conditions a 4 mm medial parapatellar incision was performed at the right knee and the patella was dislocated laterally. After drilling a hole (Ø = 0.5 mm) into the intracondylar notch a distally flattened 24 G needle was implanted intramedullary. The patella was then repositioned over the knee joint and the muscles and skin were sutured separately. Then the middle of the femur was exhibited through a lateral approach and an osteotomy was created in the middle of the femur, using a gigly-wire-saw [13], [14]. Wound closure completed the operative procedure.

### Pharmaceutical intervention

Forty-eight mice were treated daily with the ARBs Telmisartan (Boehringer-Ingelheim, Germany) (30 mg/kg) in their drinking water. An additional 48 mice served as controls, given only drinking water. Animals were killed after 2, 5 and 10 weeks by cervical dislocation. Fracture healing was evaluated by biomechanical (n = 8 each group) and by by histomorphometric and immunohistochemical anlysis (n = 8 each group). Additional animals were killed after 5 and 10 weeks for micro-CT analysis (n = 8 each group).

### Radiography analysis

At the end of the 2, 5 and 10 weeks post-fracture observation periods, the mice were anesthetized (xylazine and ketamine, same dosage as above), and ventro-dorsal radiographs of the healing femora were taken. X-rays were taken by a DMR+ Mo target mammography machine (18 kV, 160 mAs, GE, USA). The callus diameter was measured in relation to the femora diameter (%).

### Biomechanical analysis

The fractured and nonfractured femurs were subjected to torsional testing to assess biomechanical properties. Proximal and distal ends were placed into device pots and embedded using polymethylmethacrylate (PMMA). The bones were then mounted on a commercial material testing system (Instron, Norwood, MA, USA). Applying a gradually increasing angle (0.15 s-1), parameters of the fractured femora are given in percent of the corresponding unfractured contralateral femora: Peak torque at failure (%), Peak rotation angle at failure (%), Torsional Stiffness (%).

### Histomorphometric analysis

For histomorphometric analysis, the healed femora bones were fixed in 4% phosphate-buffered formalin for 24 h, decalcified in 13% EDTA solution for 2 weeks and then embedded in paraffin. Longitudinal sections of 5 um thickness were stained with hematoxylin and eosin. The central slide of each specimen was analyzed at a magnification of 5× (Leica Instruments GmbH, Germany). In adherence to the recommendations of the American Society of Bone and Mineral Research (ASBMR) [15] the following histomorphometric parameters were calculated: total callus area in relation to femoral bone diameter at the fracture gap [Cl.Ar B.Dm^−1^(%)], bone callus area (mineralized bone, osteoid and bone marrow) in relation to total callus area [B.Cl.Ar Cl.Ar^−1^ (%)], cartilaginous callus area in relation to total callus area [Cg.Cl.Ar Cl.Ar^−1^ (%)] and fibrous callus area in relation to total callus area [Fb.Cl.Ar Cl.Ar^−1^ (%)]

### Immunohistochemical analysis

Immunohistochemical staining for proliferating cell nuclear antigen (PCNA) and VEGF was used. Sections were deparaffinized using xylene and rehydrated using graded alcohols. The endogenous peroxidase was quenched using 3% hydrogen peroxide for 20 min. Antigen retrieval was achieved by microwaving (10 min; 700 W) specimens in citrate buffer (pH 6.0). After blocking unspecific binding sites with PBS and goat normal serum (30 min; room temperature), sections were incubated overnight with mouse monoclonal anti-PCNA (1∶50 PBS; Sigma, St. Louis, MO, USA)) or with mouse monoclonal anti-VEGF (1∶50 PBS; Sigma, St. Louis, MO, USA) antibodies at room temperature. Peroxidase-conjugated goat anti-mouse antibodies (1∶100; Millipore, Temecula, CA, USA) were used as secondary antibodies (incubation for 1 h at room temperature). DAB (Dako Cytomation) served as the chromogen and Mayer's hemalum as the counterstain. The immunohistochemical data were qualitatively assessed.

### Micro-computerized tomography

Micro-CT measurement was performed using a desktop micro-CT system (GE Healthcare, USA). Bones were thawed at room temperature, and excess tissue was removed. The scanning protocol was set at X-ray energy settings of 80 kV and 80 μA, and the sample was scanned over one entire 360°rotation, with an exposure time of 3000 ms/frame. A constrained 3-D Gaussian filter was used to partly suppress the noise in the volumes. The high and low radio-opacity mineralized tissues were differentially segmented by a two-level global thresholding procedure [16], [17].The callus volume of interest (VOI) was defined as the newly-formed bone tissues; the medullary canal volume and the original bone tissue were excluded from evaluation according to previous reports [18], [19]. After segmentation, bone volume/tissue volume (BV/TV) and average cross-sectional area (CsAr) were quantified within the VOI.

### Statistical analysis

All data were presented as the mean with their standard deviation (means±SD) in the text and the tables. Statistical analyses were conducted using the statistics package SPSS 16.0 software (SPSS Inc., Chicago, IL, USA). Differences among treatment groups were tested by one-way analysis of variance (ANOVA). If significant differences were indicated, differences between the means of two groups were tested by Fisher's protected least significant difference (PLSD). Observations were considered to be statistically significant at P<0.05.

## Results

### Radiological analysis

**Figure 1:**
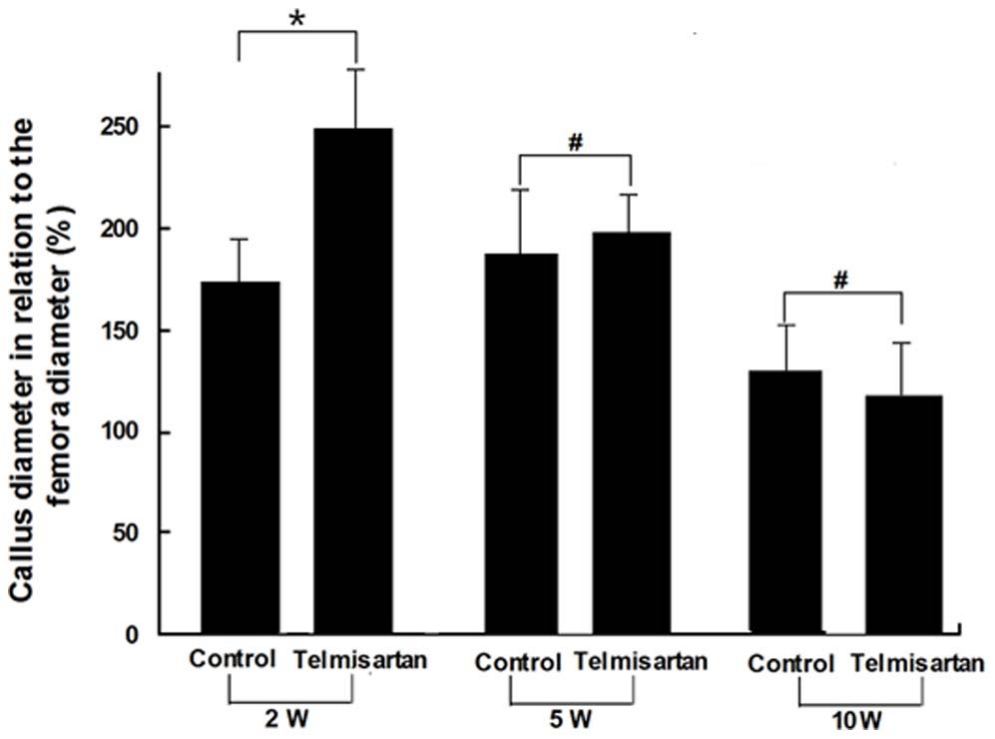
Radiological analysis of callus diameter in relation to the diameter of the femur at the fracture site after 2, 5 and 10 weeks post-fracture. All data are given as means±SD. * P<0.05 versus corresponding values of the vehicle-treated controls. # P>0.05 versus corresponding values of the vehicle-treated controls.

Radiological analyses at 2 weeks after fracture demonstrated that the diameter of the callus in the telmisartan treated animals was significantly increased when compared with that of vehicle treated controls. After 5 weeks the callus size showed no difference between the groups, whereas after 10 weeks the callus in the control animals was greater than in telmisartan treated animals. In telmisartan-treated animals, the relative callus size decreased continuously from weeks 2 to 10, while in control animals, it had increased to maximum values by week 5 and it then significantly decreased until the end of the 10 week observation period (Fig. 1).

### Biomechanical analysis

At 2 weeks post-fracture, biomechanical analysis showed a significantly greater peak torque at failure and a higher torsional stiffness in telmisartan-treated animals compared with controls (P<0.05). The peak rotation angle did not differ between the two groups. At 5 weeks post-fracture, no significant differences in maximal torque, rotation angle and torsional stiffness could be detected between telmisartan-treated animals and vehicle-treated controls. After 10 weeks post-fracture, both groups showed complete bone healing, as indicated by a torsional stiffness of almost 100% of the contralateral femur in both groups (Table 1).

**Table 1:** Biomechanical analysis of the callus of telmisartan-treated and vehicle-treated animals at 2, 5 and 10 weeks after fracture

	2 weeks			5 weeks			10 weeks		
	Control	Telmisartan	P	Control	Telmisartan	P	Control	Telmisartan	P
Peak torque [%]	16.7±4.2	31.4±5.1	0.03	60.5±13.4	78.2±16,9	0.26	76.2±11.5	81.6±16.2	0.57
Peak rotation angle [%]	90.4±13.9	79.1±15.2	0.27	83.3±15.7	67.1±14.6	0.47	75.3±10.3	84.2±13.8	0.61
Torsional stiffness [%]	19.5.±7.3	37.7±8.6	0.04	104.6±16.1	130.4±25.7	0.34	142.5±16.4	149.4±18.5	0.73

Data are mean followed by standard deviation.

### Histomorphometric analysis

**Figure 2:**
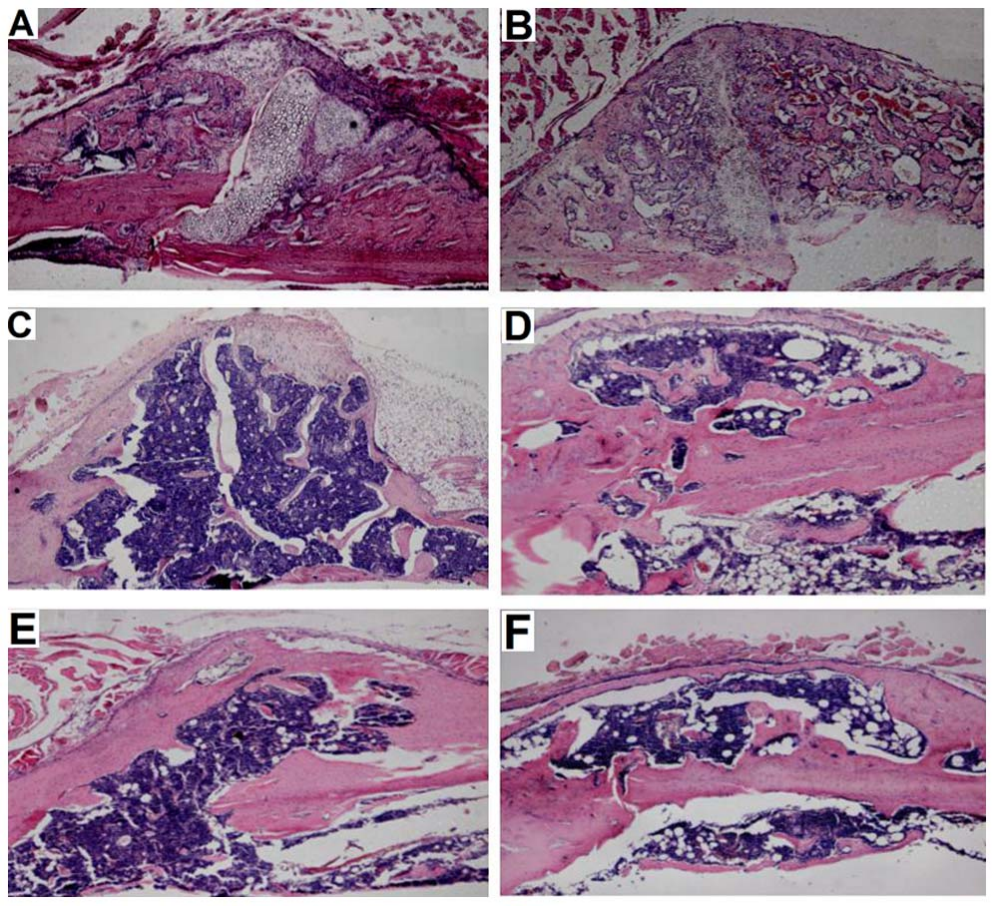
Longitudinal sections of the callus of femora at 2 (A and B), 5 (C and D) and 10 (E and F) weeks post-fracture. A, C and E show the callus of vehicle treated controls, B, D and F that of telmisartan-treated animals.

At 2 weeks after fracture, telmisartan-treated animals showed some more cartilage and markedly less fibrous tissue. Of interest, we observed a significant (P<0.05) reduction of the gap width from weeks 2 to 5 in the telmisartan-treated animals when compared with vehicle treated controls. This resulted in a significantly smaller gap at week 5 after fracture in telmisartan-treated animals. At 10 weeks after fracture, cartilaginous and fibrous tissue had disappeared in almost all samples of both groups (Fig. 2, Table 2).

**Table 2:** Histomorphometric analysis of the callus of telmisartan-treated and vehicle-treated animals at 2, 5 and 10 weeks after fracture

Histomorphometry	2 weeks			5 weeks			10 weeks		
	Control	Telmisartan	P	Control	Telmisartan	P	Control	Telmisartan	P
Cl.Ar B.Dm^−1^ (%)	167.8±13.7	236.5±21.6	0.01	183.2±16.1	201.7±15.4	0.06	148.5±11.8	131.9±9.4	0.75
B.Cl.Ar Cl.Ar^−1^ (%)	40.6±8.6	53.7±9.1	0.56	84.8±13.6	93.1±10.4	0.62	100.0±0.0	100.0±0.0	1.00
Cg.Cl.Ar Cl.Ar^−1^ (%)	16.3±5.2	35.7 ±7.9	0.21	3.1±1.2	1.5±0.4	0.57	0.0±0.0	0.0±0.0	1.00
Fb.Cl.Ar Cl.Ar^−1^ (%)	42.5±10.4	11.8±3.7	0.03	12.5±4.6	5.3±1.8	0.63	0.0±0.0	0.0±0.0	1.00

Data are mean followed by standard deviation.

The results of the histomorphometric analysis showed that periosteal callus formation was significantly greater in telmisartan-treated animals at 2 and 5 weeks when compared with vehicle treated controls. This was indicated by a significantly greater relative callus diameter. Accordingly, we observed also a significantly (P<0.05) greater periosteal callus area after 2 and 5 weeks. These differences were completely lost after 10 weeks. At this time point, all animals showed complete bone bridging of the osteotomy (Table 2).

### Immunohistochemical analysis

**Figure 3:**
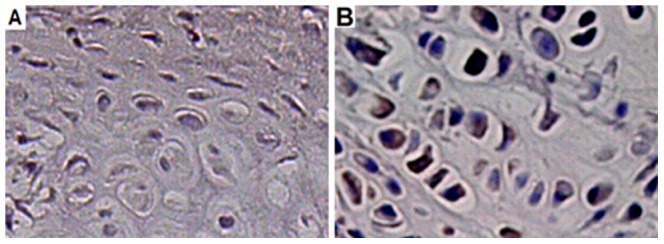
Immunohistochemistry of cell proliferation as indicated by proliferating cell nuclear antigen (PCNA) expression within the callus of a control animal (A) and a telmisartan-treated animal (B) at 2 weeks post-fracture.

**Figure 4:**
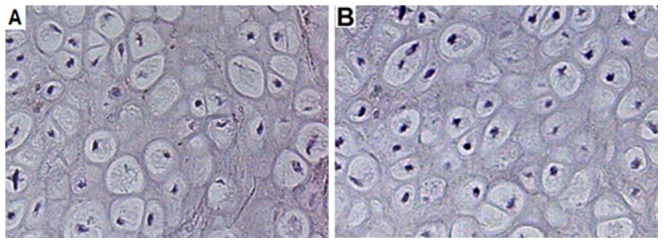
Immunohistochemistry of vascular endothelial growth factor (VEGF) expression within the callus of a control animal (A) and a telmisartan-treated (B) at 2 weeks post-fracture.

Staining for PCNA was found in osteoblasts, periosteal cells and endothelial cells at 2 weeks post-fracture. There was an increased fraction of PCNA-positive cells in telmisartan-treated group compared with vehicle-treated controls. At 2 weeks post-fracture, VEGF-positive staining was found in hypertrophic chondrocytes. The quantitative analysis also indicated a higher fraction of positive cells in telmisartan treatment animals (21.7±3.5%) compared with vehicle-treated controls (3.7±3.7%) (Fig. 3 and Fig. 4).

### Micro-CT analysis

**Figure 5:**
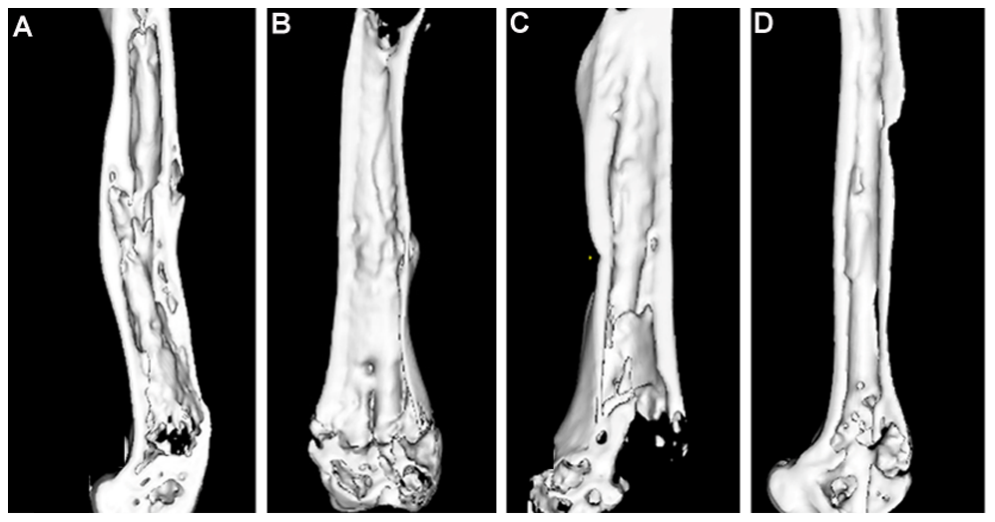
Representative micro-CT slices after 5 and 10 weeks of fracture healing in a control and a telmisartan-treated animal. A, C for the vehicle-treated group and B, D for the telmisartan-treated group.

Micro-CT analysis showed greater amount of bony callus was found in the telmisartan-treated animals than in the vehicle-treated controls at 5 weeks post-fracture, which demonstrates the anabolic effect of telmisartan during the early period of fracture healing. After 10 weeks the callus size showed no difference between the groups (Fig. 5 and Table 3).

**Table 3:** Micro-CT analysis of the callus of telmisartan-treated and vehicle-treated animals at 5 and 10 weeks after fracture

	Control	Telmisartan	P
5 weeks post-fracture (n = 5 per group)			
BV/TV(%)	70.2±8.6	85.4±7.9	< 0.01
CsAr(mm^2^)	7.3±1.2	9.2±1.4	0.03
10 weeks post-fracture (n = 5 per group)			
BV/TV(%)	98.5±7.1	96.4±9.3	0.83
CsAr(mm^2^)	5.6±0.8	5.2±0.9	0.72

Data are mean followed by standard deviation. BV/TV bone volume over total volume, CsAr average cross-sectional area.

The results of the fractured femur (Table 3) from the Micro-CT evaluation were expressed as BV/TV and CsAr. At 5 weeks post-fracture, telmisartan-treated group significantly increased the values of BV/TV by 21.7% and CsAr by 26.0% (P<0.05) compared to the vehicle-treated controls. After 10 weeks, the values of BV/TV and CsAr showed no difference between the groups (P>0.05).

## Discussion

In the present study, we analyzed the effect of telmisartan on the process of fracture healing in mice. The results point to a new possibility in the treatment of fractures. We demonstrate for the first time that telmisartan can improve fracture repair, as indicated by a signicantly increased bone formation compared to vehicle-treated controls.

Osteoporosis and atherosclerosis, two multifactorial and degenerative entities, are major public health problems. These diseases accompany the aging process and share common risk factors [20]. A correlation exists between osteoporosis and atherosclerosis regardless of age, body mass index (BMI) and cardiovascular risks. Cases of low BMD of the hip have a higher risk of cardiovascular disease (CVD) mortality in both genders [21]. Postmenopausal women with atherosclerotic changes of the abdominal aorta have a significantly decreased value of BMD at the lumbar spine and hip, as well as a higher incidence of fatal and non-fatal coronary events [22]. Pathophysiological mechanisms connecting atherosclerosis and osteoporosis are complex and can be dependent or independent of the production of vitamin D [23]. Hyperproduction of inflammatory markers such as C-reactive protein, interleukin-1 (IL-1), interleukin-6 (IL-6) and tumour necrosis factor-α (TNF-α) can be considered risk factors and some are directly related to the severity of atherosclerosis [24].

The renin-angiotensin system (RAS) is an endocrine system that governs body fluid and electrolyte balance and blood pressure [1]. In the classic endocrine RAS, angio-tensinogen produced in the liver is sequentially cleaved by peptidases to form the biologically active octapeptide angiotensin II (AngII). Renin produced by the juxtaglo-merular apparatus of the kidney and secreted into the circulation cleaves angiotensinogen to the inactive deca-peptide angiotensin I, which is cleaved by angiotensin-converting enzyme (ACE) to generate AngII. The initial reaction between the enzyme renin and the substrate an-giotensinogen is the rate-limiting step of the RAS, for which strict species specificity exists. The RAS has been an important target of antihypertensive drugs, especially ACE inhibitors and angiotensin receptor blockers (ARBs) [3], [4].

The ACE is a key component of the RAS, generating Ang II as the main effector molecule. Different in vitro studies have shown that Ang II can be synthesized by osteoblastic cells through ACE and that Ang II influences osteoblastic cell functions through specific receptor binding [25]–[27]. Ang II exerts its biological effects mainly by two receptors, the AT1 and AT2 receptors. Ang II has a multitude of biological effects in different tissues and influences inflammation, angiogenesis, cell proliferation, cell differentiation and apoptosis. Accordingly, the RAS has been shown to significantly influence tissue remodelling in various tissues [1].

Recent clinical studies indicated that beta blockers and anti-hypertension drugs would reduce the risk of bone fractures in the elderly populations [5]–[7]. This suggests a possible link between vascular and skeletal systems. Renin angiotensin system (RAS) is operating not only systemically but also locally in several tissues, and bone microenvironments have been studied in this regard [8]–[9]. Osteoblasts and osteoclasts express angiotensin II type 1 receptor in cell cultures, suggesting the existence of local RAS in bone [9], [25], [28]. One study showed that activation of RAS induces not only hypertension but also osteopenia with microstructural deterioration, reminiscent of osteoporosis, suggesting that aberrant activation of RAS may contribute to the co-occurrence of the hypertensive disorders and osteoporosis, which is often seen with advancing age [29].

It has been proposed that inhibition of RAS with ACE inhibitors or ARBs has beneficial effects beyond those resulting from lowering blood pressure alone, such as renoprotective effects and cardiovascular outcomes [30]. Epidemiological studies indicate that patients who have undergone stroke have an increased risk of hip fracture [31]–[32]. However, one study using rats reported that exogenous Ang II administration suppressed bone mass levels through AT1 receptor in animals subjected to ovariectomy and five-sixths nephrectomy [9]. Also, a study of mice with complete NO synthase deficiencies revealed that increased bone mass by genetic disruption was normalized by AT1 blockade [29].

ARBs are widely used in cardiovascular medicine, it is of particular interest to elucidate how these drugs may interfere with bone homeostasis. A clinical study reported that angiotensin-receptor blocker use was associated with a large and significant reduction. They believed that the fracture reduction associated with angiotensin-receptor blockers suggests a possible mechanism for affecting fracture risk [5]. In addition, a previous study using spontaneous hypertensive rats revealed that AT1 blocker inhibited the reduction of bone loss, but no effect of AT2 was observed in fracture risk [33].

In the present study, systemic treatment with telmisartan during fracture healing resulted in an increased callus formation after 2 and 5 weeks. This was most probably due to an increase of cell proliferation in the periosteal callus, as indicated by an increased fraction of PCNA-positive cells and VEGF-positive cells in telmisartan-treated group compared with vehicle-treated controls. The improved periosteal callus formation resulted in an accelerated healing process with an earlier histological bridging of the fracture gap and an advanced remodelling after 10 weeks. The improved histological healing of the fractures after telmisartan treatment is further supported by the results of our biomechanical analysis, which demonstrated a greater torque to failure and a higher torsional stiffness after 2 and 5 weeks in telmisartan-treated animals. Of interest, after 2 weeks of fracture healing, the callus of telmisartan-treated animals showed less fibrous tissue compared with controls.

It is well known that VEGF is one of the key growth factors promoting endochondral ossification also during secondary fracture healing. This stage of fracture healing consists of terminal chondrocyte differentiation and cell death, extracellular matrix degradation and calcification as well as angiogenesis and osteogenesis [34]. VEGF has been reported to be expressed in terminally differentiating chondrocytes, coincidently with the onset of vascularization and subsequent ossification of the callus [35]. Hence, the positive action of telmisartan on fracture repair at 2 weeks post-fracture might be due to an inhibition of VEGF expression in hypertrophic chondrocytes as shown by immunohistochemical analysis. On the other hand, the increased amount of VEGF expression might also be a consequence of altered cell proliferation and differentiation as indicated by the increase PCNA content in the callus of telmisartan-treated animals when compared with vehicle-treated controls.

This study has one major limitation. Our fracture model was established based on a surgical osteotomy in contrast with a closed fracture model, the latter of which better describes common clinical fractures. However, this model enabled us to make a consistent fracture line, which is essential for making reliable and accurate evaluations both mechanically and histologically.

In summary, we demonstrate in the current study that telmisartan could promote fracture healing in a mice model via increasing mechanical strength and improving microstructure. The most mechanism is probably by an increase of cell proliferation and neovascularization associated with a decreased VEGF expression in hypertrophic chondrocytes. Considering the increasing administration of telmisartan in hypertension patients, the results of this study suggest that physicians could prescribe telmisartan to fracture patients who have hypertension, for the purpose of not only for hypertension, but also promoting fracture healing.
